# Epoxyeicosanoids prevent intervertebral disc degeneration *in vitro* and *in vivo*

**DOI:** 10.18632/oncotarget.14389

**Published:** 2016-12-30

**Authors:** Jing Li, Hanfeng Guan, Huiyong Liu, Libo Zhao, Li Li, Yong Zhang, Peng Tan, Baoguo Mi, Feng Li

**Affiliations:** ^1^ Department of Orthopaedic Surgery, Tongji Hospital, Tongji Medical College, Huazhong University of Science and Technology, Wuhan, Hubei, China; ^2^ Department of Radiology, Tongji Hospital, Tongji Medical College, Huazhong University of Science and Technology, Wuhan, Hubei, China

**Keywords:** epoxyeicosatrienoic acids, intervertebral disc degeneration, nucleus pulposus cells, NF-κB, Pathology Section

## Abstract

Intervertebral disc (IVD) degeneration is considered a common cause of low back pain. In the degenerating IVD, the production of pro-inflammatory cytokines, including IL-1 and TNF-α, progressively increases, contributing to the degenerative process. Epoxyeicosatrienoic acids (EETs), synthesized from arachidonic acid by cytochrome P450 enzymes, act as autocrine and paracrine effectors in regulating inflammation, cardiovascular functions, and angiogenesis. EETs were shown to be especially potent promoters of tissue regeneration. Considering their anti-inflammatory and anti-catabolic potential, we investigated whether EETs can influence IVD degeneration. We found that 14,15-EET protected rat nucleus pulposus (NP) cells against death induced by treatment with H_2_O_2_and TNF-α *in vitro*. At the molecular level, 14,15-EET significantly inhibited the NF-κB pathway, which plays essential roles in the degeneration and survival of NP cells. As a result, 14,15-EET efficiently prevented the matrix remodeling response of NP cells to TNF-α. Using a needle-punctured rat tail model, the influence of 14,15-EET on IVD degeneration *in vivo* was evaluated using radiographs, magnetic resonance images (MRI), and histological analysis. We observed that 14,15-EET prevented IVD degeneration. Our findings demonstrated that 14,15-EET can enhance the survival of NP cells and inhibit IVD degeneration. The EET pathway may be a novel therapeutic target against IVD degeneration.

## INTRODUCTION

Low back pain and neck pain, two of the most common reasons patients seek medical care, have a combined lifetime prevalence of 54-80% [1]. According to estimates from a recent report, low back pain affects 632 million people worldwide and represents the leading cause of disability [2]. Degeneration of intervertebral discs (IVDs) is generally considered to be the main cause of low back pain and neck pain [3]. IVDs are located between adjacent vertebrae in the vertebral column. Each disc is composed of the peripheral annulus fibrosus (AF) and the central nucleus pulposus (NP). The AF is a fibrous tissue with concentric lamellae that are rich in collagen fibrils [4]. The NP has a high content of the proteoglycan aggrecan (Agg), which provides the disc with the ability to resist compressive loads [5]. IVD homeostasis relies on a delicate balance among cells, the extracellular matrix, biomechanical stress, the endplate, and other factors. Signs of IVD degeneration can be observed in almost all adults as they age. Degeneration may begin as early as the first decade of life, when there is a gradual transition from notochordal cells to chondrocyte-like cells in the NP [6]. These chondrocyte-like cells produce more collagen type I (Col I) and less water-attracting proteoglycans and collagen type II (Col II), leading to matrix remodeling characterized by a decrease in proteoglycan synthesis and an increase in Col I. This remodeling impairs the ability of the NP to retain water and maintain its structure and composition under compressive forces. Inside the degenerating IVD, the production of pro-inflammatory cytokines, including IL-1 and TNF-α, increases progressively [3, 7]. These cytokines promote matrix remodeling that is mainly mediated by two families of enzymes: matrix metalloproteinases (MMPs) and disintegrin and metalloproteinases with thrombospondin motif proteins (ADAM-TS). As IVD degeneration progresses, pro-inflammatory cytokines also promote neurovascular in-growth and induce pain response [3].

Epoxyeicosatrienoic acids (EETs), including 5,6-EET, 8,9-EET, 11,12-EET, and 14,15-EET, are produced from arachidonic acid by cytochrome P450 enzymes. These signaling molecules have both autocrine and paracrine actions. EETs have potent anti-inflammatory properties through mechanisms including suppression of NF-κB activation [8]. We have previously shown that EETs suppress osteoclastogenesis and prevent bone loss both *in vitro* and *in vivo* by suppressing NF-κB activity, reactive oxygen species (ROS) production, and levels of inflammatory cytokines IL-1 and TNF-α [9]. EETs inhibit apoptosis by modulating PI3K/Akt and MAPK signaling pathways. Recent evidence highlighted EETs as potent tissue regeneration promoters [10]. EETs are able to accelerate the regeneration of multiple organs and tissues, including the liver, kidney, and lung, and they promote wound healing, corneal neovascularization, and retinal vascularization *in vivo* [10]. Furthermore, EETs have therapeutic effects on pain [11, 12]. Therefore, multiple clinical trials aimed at harnessing the anti-inflammatory and pro-regenerative properties of EETs are underway [13, 14]. Considering the need for novel strategies for restoring IVD anabolism and preventing degeneration, we used both *in vitro* and *in vivo* models to investigate whether EETs can inhibit IVD degeneration and elucidate the molecular mechanisms involved in this process.

## RESULTS

### 14,15-EET protects NP cells from hydrogen peroxide cytotoxicity *in vitro*

**Figure 1:**
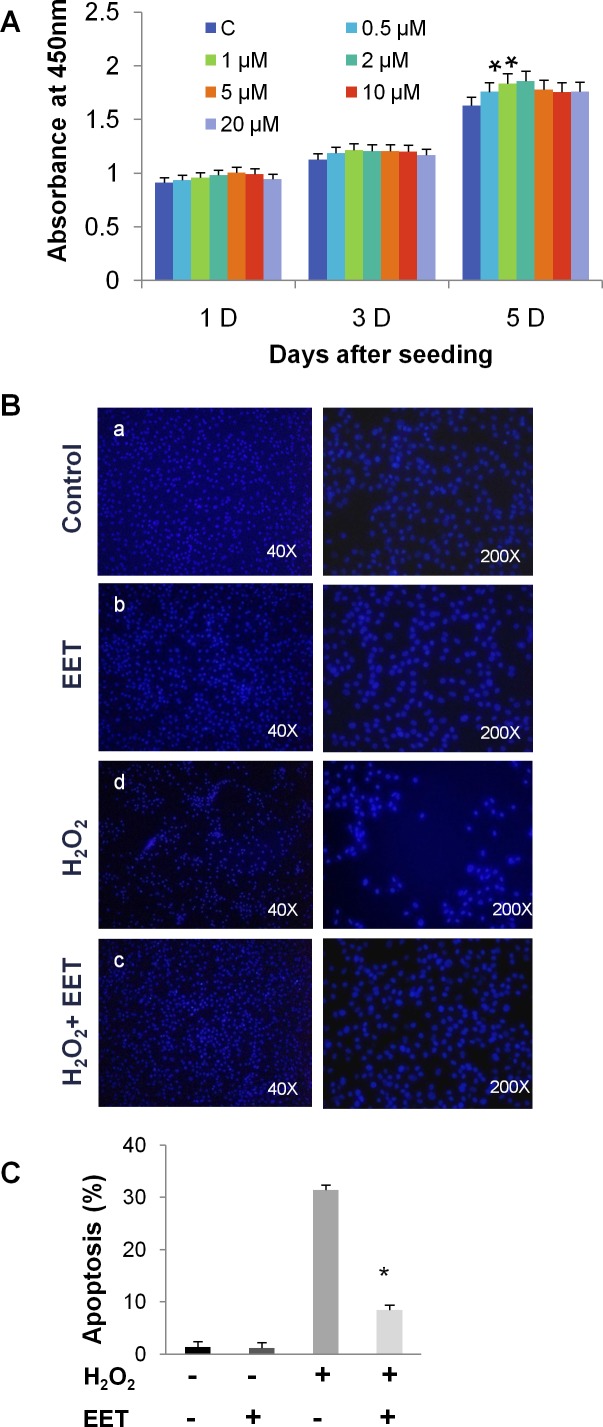

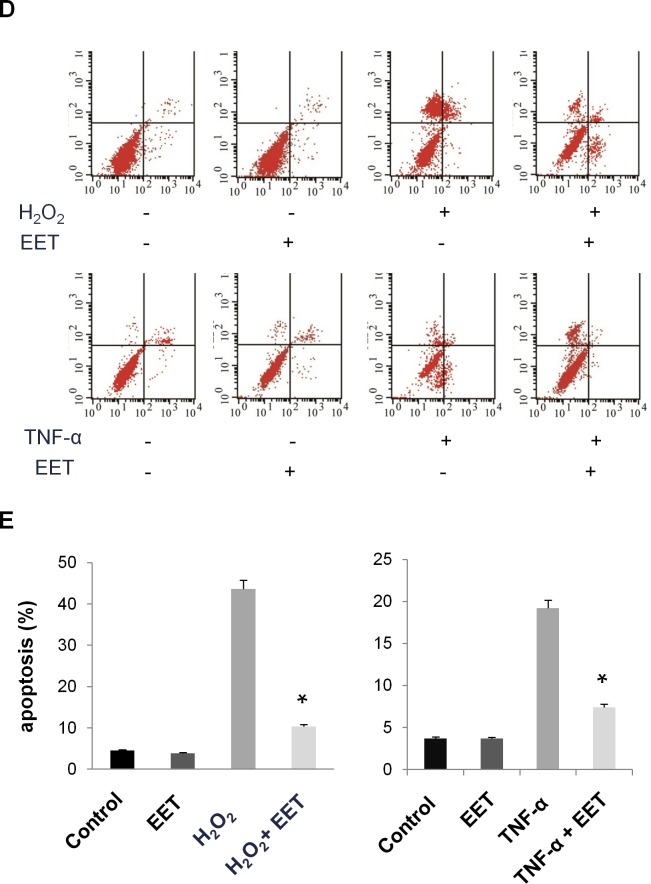
EET protects cultured NP cells from hydrogen peroxide- and TNF-α-induced cytotoxicity **A**. NP cells were seeded in 96-well plates at a density of 1×10^3^ cells per well and treated with EET at the indicated concentrations. Cell viability was measured by a CCK-8 kit. * *p* < 0.05 (compared with samples without EET treatment). **B**. H_2_O_2_ treatment (20 μM, 4 hours) induced cell detachment from the plates. EET efficiently prevented the deleterious effects of H_2_O_2_. **C**. Apoptosis of H2O_2_-treated NP cells was measured by annexin V/PI staining. Cells staining positive for either annexin V or PI were considered apoptotic or necrotic. Data represent means ± SD of three independent experiments. * *p* < 0.05 (compared with samples treated with H_2_O_2_ alone). **D**., **E**. Apoptosis of H_2_O_2_- or TNF-α treated NP cells measured by annexin V/PI staining. After treatment, floating cells and adhered cells were collected separately and pooled for annexin V/PI staining. **E**. Data represent mean ± SD of three independent experiments. * *p* < 0.05 (compared with samples treated with H_2_O_2_ or TNF-α alone).

We first tested the influence of EET, at concentrations ranging from 0 to 20 μM, on the proliferation of cultured NP cells. After 5 days of treatment in monolayer culture, CCK-8 assays demonstrated that EET (from 0.5 to 5 μM) was not harmful to NP cells (Figure 1A). Moreover, we observed a slight but statistically significant promotion of proliferation at concentrations of 1 and 2 μM. For all subsequent *in vitro* experiments, we treated cells with 2 μM 14,15-EET. Oxidative stress with ROS overproduction induces the apoptosis of NP cells and is associated with disc degeneration [15]. We examined whether EET could protect NP cells from oxidative stress induced by hydrogen peroxide (H_2_O_2_). As expected, after H_2_O_2_ treatment (20 μM, 4 hours), a significant number of cells detached from the plate, indicating that H_2_O_2_ impaired survival. Remarkably, treatment with EET efficiently prevented the deleterious effects of H_2_O_2_ (Figure 1B and 1C). Using annexin V and PI staining, we found that H_2_O_2_ or TNF-α treatment induced massive apoptosis, and EET protected NP cells from H_2_O_2_ and TNF-α cytotoxicity (Figure 1D and 1E).

### 14,15-EET prevents TNF-α induced matrix destruction

**Figure 2:**
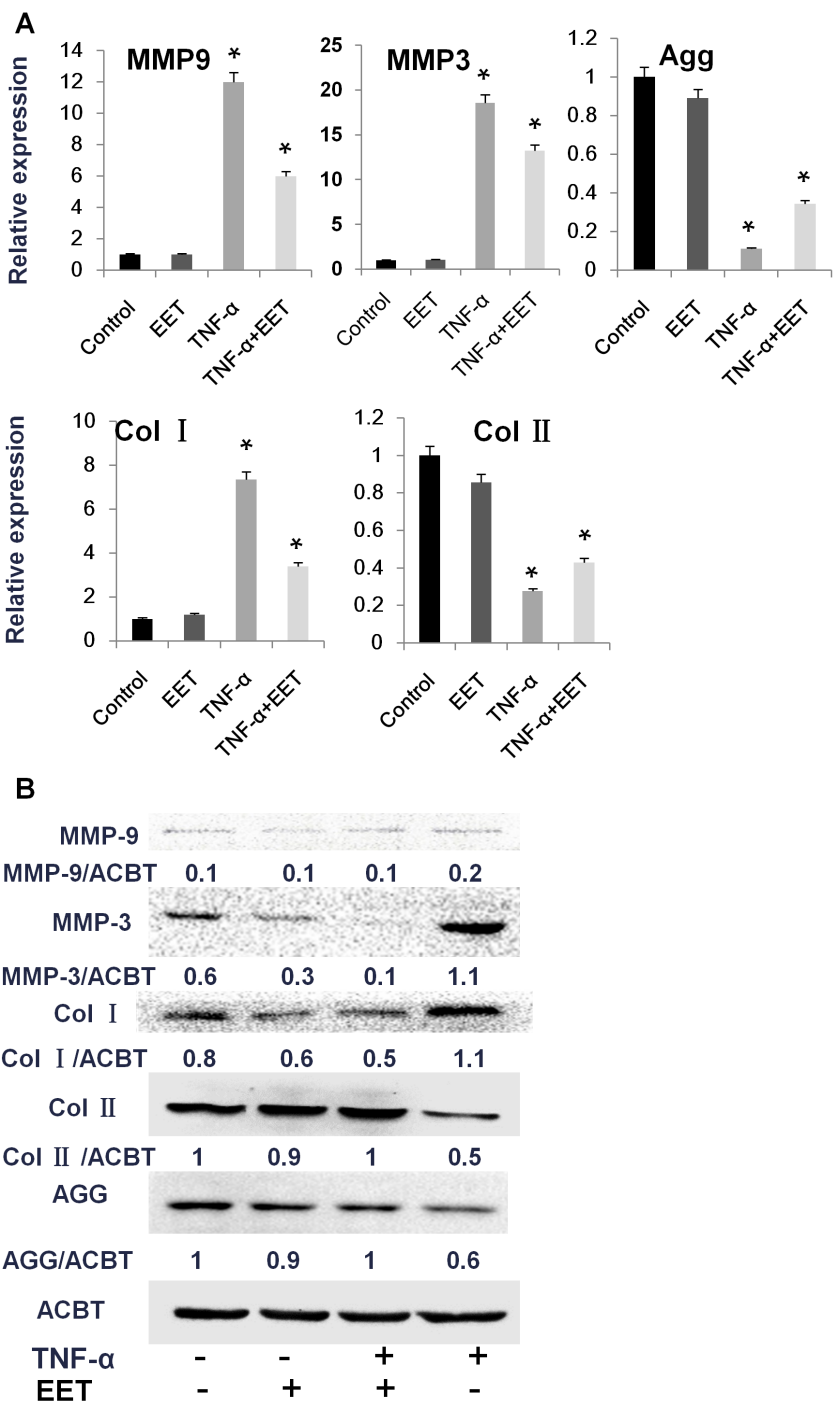
EET prevents TNF-α-induced matrix destruction NP cells were cultured in complete medium for 1 day, then treated with TNF-α (50 ng/ml) in serum-free media for 6 hours. Thereafter, EET was added (2 μM), and cells were incubated for an additional 3 days. Untreated control groups as well as TNF-α- or EET-treated groups were included in the experimental setup. Subsequently, cells were collected for RNA and protein preparation. **A**. Expression of target genes was determined by quantitative PCR and calculated in relation to internal control gene GAPDH by the comparative Ct method. * *p* < 0.05 (compared with samples treated with TNF-α alone). Agg: Aggrecan, Col1α1: Type I collagen, Col2α1: Type II collagen. **B**. Protein expression was measured by immunoblotting. Anti-ACTB antibody was used as a loading control. The immunoblots were quantified using ImageJ software. Experiments were repeated three times with similar results.

EET is known to be a potent inhibitor of inflammation. During IVD degeneration, NP cells, AF cells, and infiltrating immune cells secrete high levels of inflammatory cytokines, especially TNF-α and IL-1β. These cytokines induce MMP expression, leading to decreased Col II and Agg and increased production of Col I [16]. We validated the protective effects of EET on TNF-α induced matrix remodeling. As expected, treatment with TNF-α significantly increased expression of MMP3 and MMP9 at the mRNA level, and the MMP3 protein was strongly upregulated (Figure 2). EET attenuated the increased mRNA expression of MMP3 and MMP9. Interestingly, at the protein level, EET almost completely prevented MMP3 expression. As a result, EET efficiently prevented the matrix remodeling response to TNF-α, at both the mRNA and protein levels. The expression patterns of Col I, Col II, and Agg in the TNF-α + EET group were similar to those in the control group (Figure 2).

### 14,15-EET inhibits the NF-κB pathway

**Figure 3:**
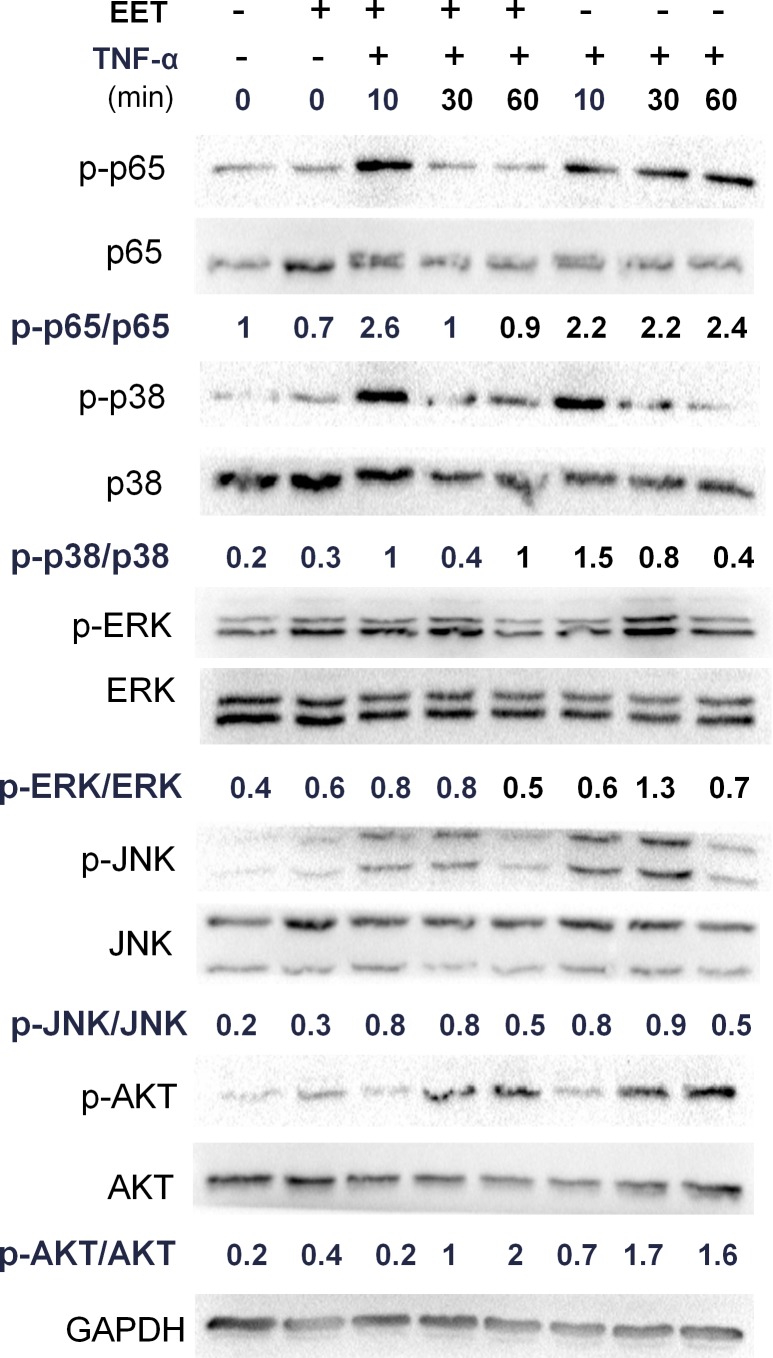
EET inhibits the NF-κB pathway NP cells were cultured in serum-free media for 8 hours. Then, the cells were treated with EET (2μM) or vehicle for 2 hours. Subsequently, TNF-α (50 ng/ml) was added, and cells were incubated for 10, 30, or 60 minutes. Cells were then collected and total protein extracted. The expressions of p-p65, p-p38, p-ERK, p-JNK, and p-AKT were detected by immunoblotting. Antibodies to GAPDH and total p65, p38, ERK, JNK, and AKT were used as loading controls. The immunoblots were quantified using ImageJ software. Experiments were performed in triplicate.

To understand the mechanisms underlying EET prevention of the matrix remodeling response, we measured the activity of major molecular pathways in EET-treated NP cells (Figure 3). As expected, TNF-α treatment activated the NF-κB pathway, as indicated by increased levels of phosphorylated p65 (p-p65), as well as MAPKs and the PI3K/AKT signaling pathway, as shown by increased phosphorylation of their core proteins. In the TNF-α-treated NP cells, p-p65 levels were highly upregulated 10 min after stimulation in both groups, irrespective of EET treatment. After 10 min, EET treatment efficiently reduced p-p65 levels. Among the three major subfamilies of MAPKs, phosphorylated p38 (p-p38) and phosphorylated JNK (p-JNK) levels showed no significant changes upon EET treatment, whereas phosphorylated ERK (p-ERK) demonstrated a slight but significant downregulation in the EET-treated cells. The PI3K/Akt signaling pathway was not significantly influenced by EET in the TNF-α-treated NP cells (Figure 3).

### 14,15-EET partially prevents puncture-induced disc degeneration

**Figure 4:**
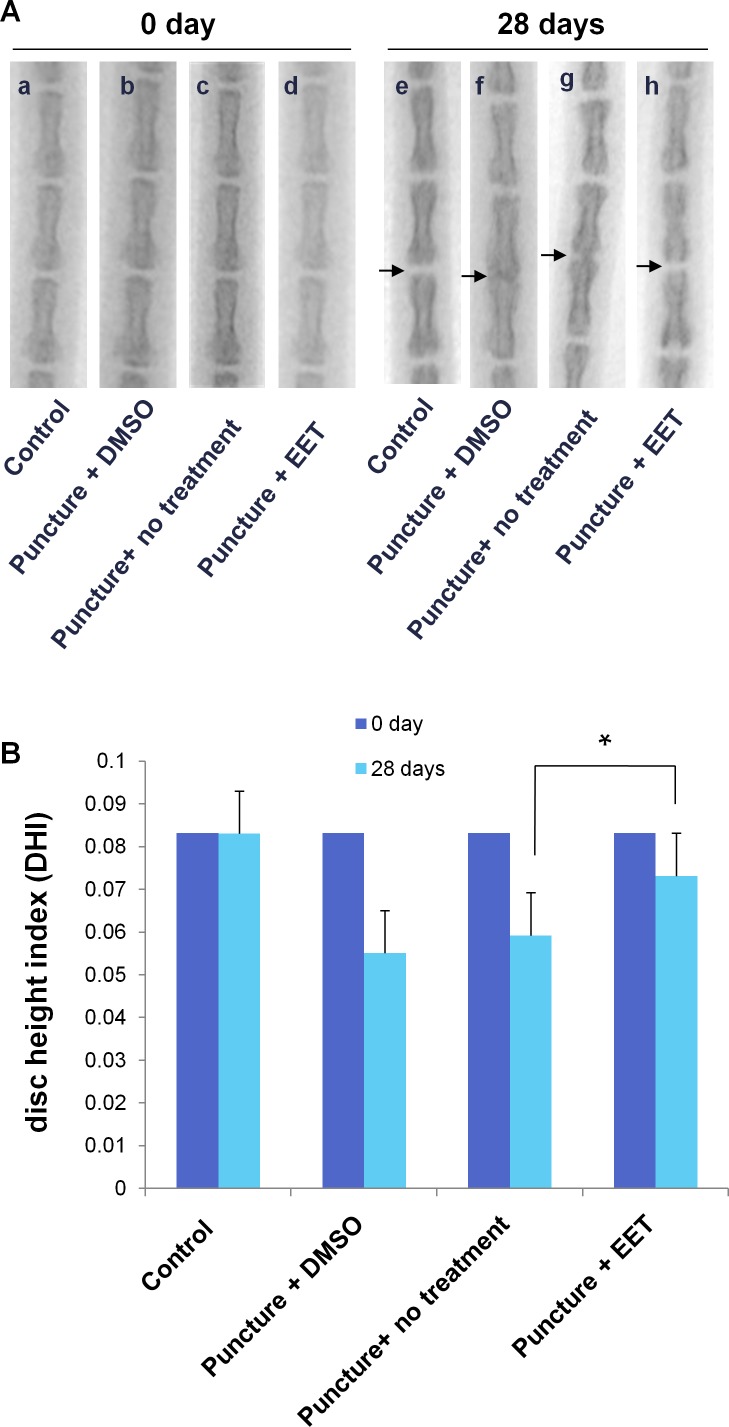
EET prevents puncture-induced disc space narrowing **A**. Radiographic images of rat tails (a-d) preoperative (0 day) and (e-h) 4 weeks after injury by needle puncture. Control: the non-punctured control group; DMSO: the DMSO (vehicle)-treated punctured group; puncture: punctured group, not treated with either EET or DMSO; EET: EET-treated punctured group. The evaluated disc spaces are indicated with arrows. Each group consisted of 10 rats (10 discs). **B**. Disc height index (DHI) was calculated preoperatively and at 4 weeks postoperative. *p < 0.05.

Encouraged by the *in vitro* results, we next tested whether EET could be used for the prevention of disc degeneration *in vivo*. We used a disc puncture procedure to model IVD degeneration in rats, which is the most commonly used method. Moreover, the disc puncture procedure is clinically relevant, as multiple diagnostic and therapeutic approaches for IVD disease involve needle puncture. After the procedure, rats were given 10 doses of EET or DMSO. Radiographic images and MRIs were taken 4 weeks after puncture and were compared with images taken before the procedure. Prior to the puncture, no signs of degeneration could be found on either the X-rays or the MRIs. Four weeks later, in the punctured groups without EET treatment, the radiographic images showed a loss of disc height or even a collapsed disc space and osteophyte formation, indicating progressive degeneration (Figure 4A). In contrast, in the EET-treated punctured group, no significant disc space narrowing or osteophyte formation was observed. After the procedure, in the DMSO-treated and untreated punctured group, the disc height index (DHI) decreased from 0.083 to 0.055 ± 0.005 and 0.0592 ± 0.005, respectively. In the EET-treated group, there was only a slight decrease in the DHI (0.073 ± 0.007), which was significantly higher than that in the punctured groups without EET treatment. Therefore, EET efficiently attenuated puncture-induced loss of disc height.

**Figure 5:**
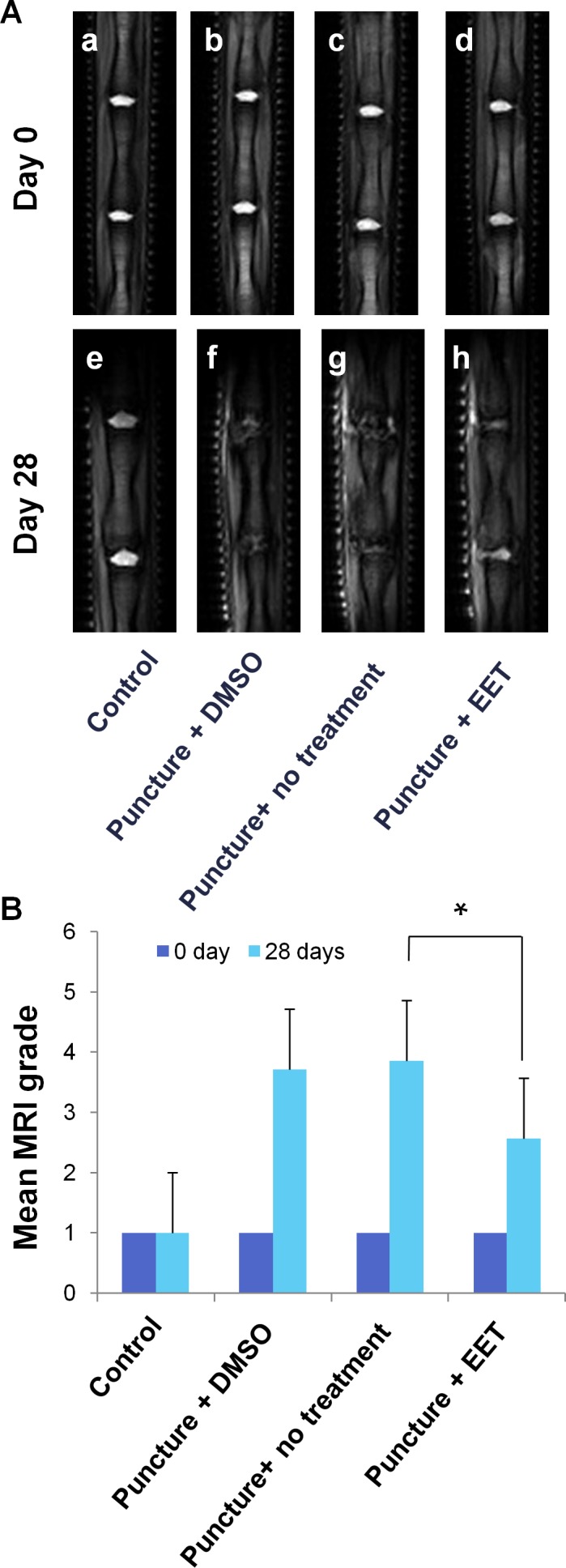
EET improves MRI disc grade **A**. T2-weighted MR mid-sagittal images of rat tails. Each group consisted of 10 rats (20 discs). **B**. Grading of disc degeneration was performed in T2 MR mid-sagittal images according to the system proposed by Pfirrmann et al. A higher grade indicates more severe degeneration. *p < 0.05.

Grading of disc degeneration was performed in T2-weighted mid-sagittal images according to the system proposed by Pfirrmann et al. [17]. Four weeks after the procedure, no evidence of degeneration was observed in the sham-operated control group. In contrast, punctured discs without EET treatment collapsed, the structure was heterogeneous, and the T2 MR signal intensity was gray to dark, suggesting severe degeneration. In the EET-treated punctured group, discs demonstrated decreased height and T2 signal intensity compared with the control group. However, the T2 signal intensity was significantly stronger and the structure was more homogenous compared with those in the punctured groups without EET treatment. In conclusion, EET significantly improved the MRI grade of degeneration (Figure 5).

**Figure 6:**
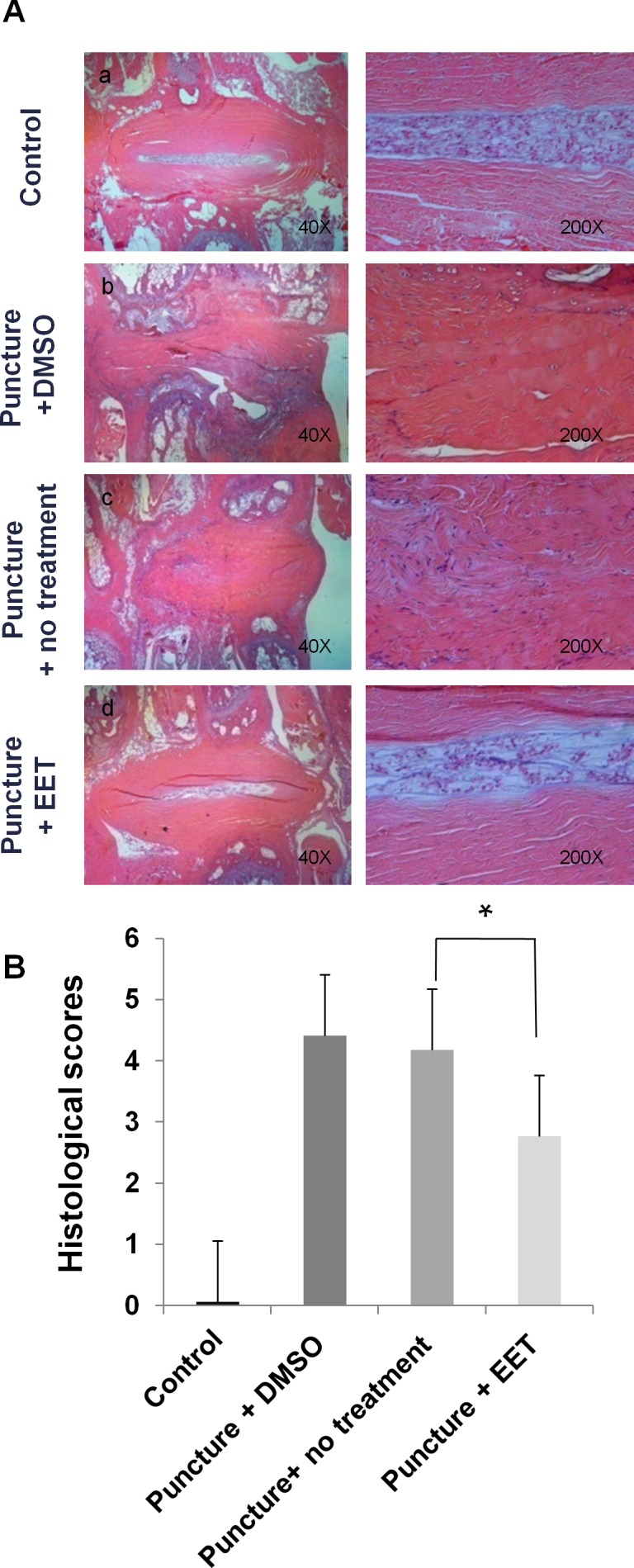
EET preserves disc structure **A**. H&E staining was performed to examine the morphology of IVDs. Four weeks after puncture, H&E staining of sagittal slices of Co5-Co6 discs was obtained for each group and magnified 40x and 200x. Each group consisted of 10 rats (10 discs). **B**. Histological grading of AF and NP. A higher score indicates more severe disc degeneration. *p < 0.05.

In the sham-operated control group, H&E staining demonstrated an intact circumferential AF, and the border between the AF and the NP was clear. In the punctured groups with DMSO or no treatment, a disorganized structure with abundant ruptured and serpentine-patterned fibers was observed in the AF. Furthermore, the border between the AF and NP was interrupted and unclear, exhibiting features of severe degeneration. In the EET-treated punctured group, the organized structure of the AF was preserved, with few ruptured and serpentine-patterned fibers, and the border between the AF and NP was readily distinguishable (Figure 6). Statistical analysis demonstrated that the histological score of the EET-treated groups was significantly lower than that of the punctured groups without EET treatment.

**Figure 7:**
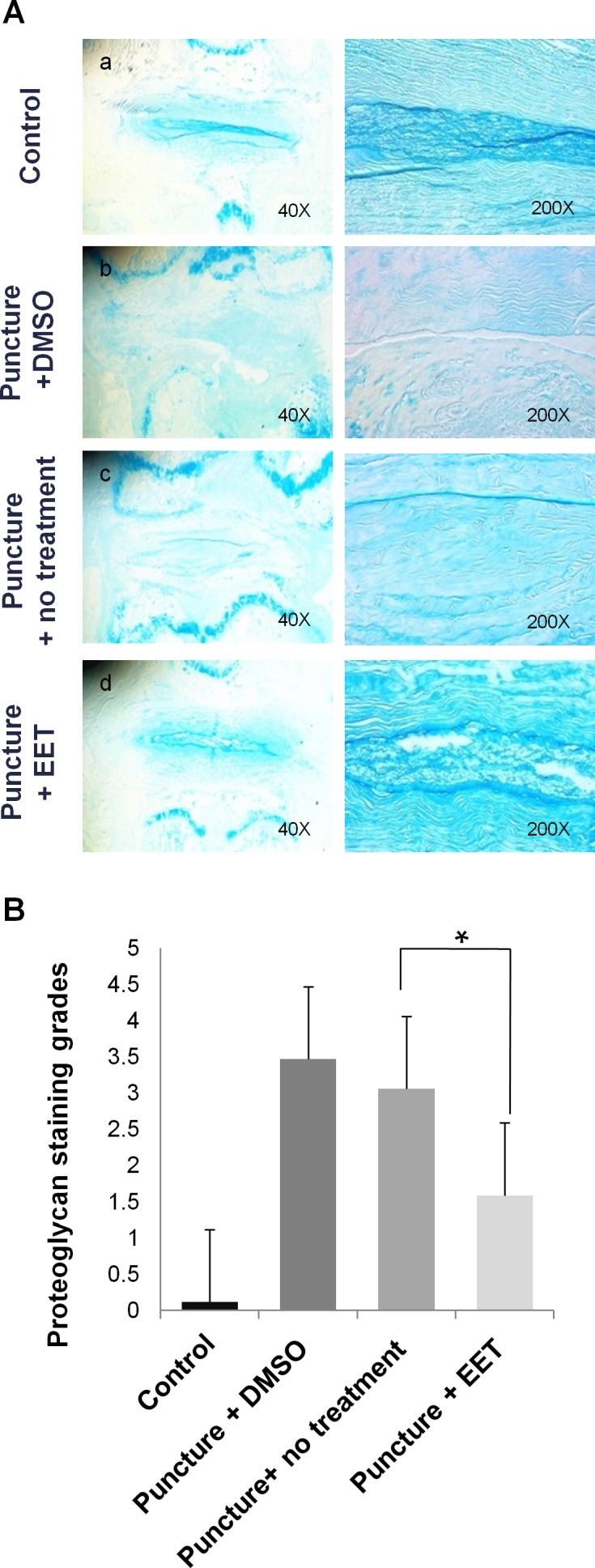
EET preserves proteoglycan density in discs **A**. Alcian blue staining was used to evaluate the density of proteoglycan in the discs. Stained images were magnified 40x and 200x. Each group consisted of 10 rats (10 discs). **B**. Proteoglycan grades of alcian blue stained sections. A higher grade indicates a more proteoglycan in the disc. *p < 0.05.

We used Alcian blue staining to evaluate the density of proteoglycan in the discs. In the sham-operated control group, deep blue staining was confined to the NP and the innermost layers of the AF; however, in the punctured groups with DMSO or no treatment, this staining pattern disappeared, and large areas of the discs stained faded to blue or very weak blue. In the EET-treated punctured group, the discs showed pronounced improvement in proteoglycan content, as demonstrated by stronger blue staining that was more similar to that of discs in the control group than to that of discs in the other punctured groups (Figure 7A). The proteoglycan staining grades were much higher in the EET-treated groups than in punctured groups without EET treatment (Figure 7B).

## DISCUSSION

In this study, we showed that EETs suppress the apoptosis and degeneration of NP cells *in vitro* and retard the process of intervertebral disc degeneration *in vivo.* At the molecular level, EETs inhibited the NF-κB pathway that plays an essential role in IVD degeneration and survival of NP cells. As a result, EET efficiently prevented the TNF-α-induced matrix remodeling response in NP cells; the expression pattern of MMPs, Col I, Col II, and Agg in the TNF-α + EET group was similar to that in the control group. In the rat tail model, EET effectively attenuated disc degeneration, as shown by multiple evaluation methods.

The treatment of back pain associated with disc disease, discogenic pain, and radiculopathic pain, is still a matter of debate, as current treatment options are not ideal. There is a need for novel therapeutic strategies for pain relief and limiting further degeneration of the IVD. The ideal treatment should exhibit a strong anti-catabolic effect, thus limiting further degeneration of the IVD. Previous studies showed that the inhibition of key inflammatory factors, including cyclooxygenase (COX) 2 [18], ROS [15], receptor activator of NF-κB ligand (RANKL) [19], and TNF-α [20, 21] leads to pain reduction and even retardation of degeneration. In this context, administering EETs or stabilizing endogenous levels of EETs may be a valid therapeutic strategy for a number of reasons. First, EETs have potent anti-inflammatory, anti-oxidative, and anti-apoptotic activities in that they inhibit multiple pathways, including ROS, RANKL, IL-1, and TNF-α [8–10, 12, 22, 23]. Second, as potent promoters of tissue regeneration [10], EETs may induce a shift toward anabolism from catabolism within the disc microenvironment. Third, EETs are effective for treating both inflammatory and neuropathic pain [11, 12]. Last but not least, pro-inflammatory cytokines secreted by degenerating discs promote the migration and activation of immune cells, further amplifying the inflammatory reaction and stimulating the infiltration of microvasculature and nerve fibers. The inhibitory effects of EET on leukocytes and the immune response [8, 23–25] could be beneficial in preventing immune cell migration and the subsequent formation of microvasculature and nerve fibers.

The NF-κB transcription factors are likely one of the major downstream pathways mediating the effects of EETs on IVD degeneration. Multiple studies have shown the repression of this pathway by EETs [8, 9, 24, 25]. Previous efforts have established NF-κB as a key mediator of IVD degeneration and a valid therapeutic target for mitigating IVD diseases [16, 26, 27]. NF-κB has been shown to be partially responsible for MMP upregulation in NP cells treated with IL-1 or TNF-α [26, 28, 29]. In the present study, both NF-κB activity and MMP3 expression were remarkably suppressed upon EET treatment, as indicated by decreased phosphorylated p65 and MMP3 protein expression, suggesting that EET treatment suppresses the NF-κB - MMP3 pathway. Previous studies have also reported attenuated MMP expression by EETs [30, 31]. In contrast, other studies have shown that EETs promote the expression of MMPs [32, 33]. It is unclear whether these contradictory results were due to the differential effects of EETs on NF-κB activity.

The present study has several limitations. First, we found that EET attenuated disc degeneration in the rat tail model. We attributed this phenomenon to the anti-inflammatory and pro-survival properties of EETs, which were reflected in our *in vitro* results. We observed a slight but statistically significant increase in the proliferation of NP cells treated with EET *in vitro*. We believe EET may promote the regeneration of NP cells *in vivo*, as the pro-regenerative potential of EETs has been previously shown in various tissues [10]. Second, degenerated IVD cells secreted multiple pro-inflammatory cytokines, including TNF and IL-1, which promote extracellular matrix degradation and lead to further degeneration as well as disc herniation and radicular pain [16]. We previously found that EET can decrease the levels of TNF and IL-1 [9]. EETs might also inhibit the secretion of these cytokines by NP cells. Validation of these issues *in vivo* was hampered by technical difficulties. Further efforts are needed to clarify these issues. Third, due to the short half-life of EETs *in vivo*, multiple injections of the reagent were given, which is not practical for clinical application. Considering the success of EETs in preventing IVD degeneration, administration of EET analogues or stabilization of endogenous levels of EETs has therapeutic potential against IVD degeneration.

EETs or sEH inhibitors, which increase endogenous EETs, stimulate the growth and metastasis of pre-existing cancers in mice [34]. sEH inhibitors, combined with COX-2 inhibitors, are commonly used to treat low back pain and inhibit tumor growth and metastasis [35]. Whether dual inhibition of sEH and COX-2 could lead to better outcomes related to IVD protection and pain relief is an intriguing future line of investigation.

In conclusion, for the first time, our study revealed the novel effects of EETs on intervertebral disc degeneration. Although our results from rat models should not be extrapolated to humans without further investigation, they are relevant to the pathogenesis and treatment of IVD degeneration that causes the ubiquitous ailment of low back pain.

## MATERIALS AND METHODS

### Reagents

EETs were purchased from Cayman (Ann Arbor, MI, USA) and were dissolved in DMSO. All four EETs were used in preliminary *in vitro* studies to evaluate their influence on NP cell proliferation as well as survival following H_2_O_2_ treatment. No significant differences were documented among the different EETs; thus 14,15-EET was used for all subsequent *in vitro* and *in vivo* studies.

### Animals

Three-month-old male Sprague-Dawley (SD) rats (250-300 g) were used. Animals were purchased from the Experimental Animal Center of Tongji Medical College (Wuhan, China). The animals were kept in ventilated filter-top cages under standard laboratory conditions and maintained on a 12:12 h light-dark cycle at a constant temperature of 24°C. Rats were fed conventional rodent chow and water *ad libitum*. All animal studies were approved by the Institutional Animal Research Committee of Tongji Medical College. The animal experiments were carried out in accordance with protocols approved by the Institutional Animal Care and Use Committee.

### Cell isolation and culture

NP tissues were isolated from adult SD rats (~ 350 g body weight). NP tissues were cut into small pieces and digested with 0.01% collagenase (Crescent Chemical, Islandia, NY, USA) at 37°C and 5% CO_2_ for 4-6 hours. The isolated cells were cultured in complete medium, consisting of DMEM/F12 (Life Technologies, Carlsbad, CA, USA) containing 15% fetal bovine serum (FBS; Life Technologies) with penicillin (100 U/ml) and streptomycin (100 μg/ml), at 37°C and 5% CO_2_.

### Cytotoxic assay of EET

Rat NP cells were seeded in a 96-well plate (1 × 10^4^/well) and incubated in culture medium overnight. Cells were then treated with various concentrations of EET. The cytotoxic assay was performed with a Cell Counting Kit-8 (CCK-8, Dojindo, Kumamoto, Japan) according to the manufacturer's protocol after 1, 3, and 5 days of culture.

### Hydrogen peroxide and EET treatment

The NP cells were cultured for 24 hours in complete medium and then treated with H_2_O_2_ (20 μM), with or without EET, in serum-free media for 4 hours. Microphotographs were taken under an Olympus BX51 microscope equipped with an Olympus DP70 digital camera (Olympus, Tokyo, Japan). Apoptosis of the NP cells was measured by annexin V/PI staining (Biolegend, San Diego, CA, USA) as previously described [36].

### TNF-α and EET Treatment

The NP cells were expanded (passage 2 to 4) and cultured (5 × 10^4^ cells/cm^2^) in complete medium for 1 day, and the cells were then treated with TNF-α (50 ng/ml) in serum-free media for 6 hours to induce an inflammatory and catabolic response. Thereafter, EET (2 μM) was added, and cells were incubated for 3 additional days. Untreated control groups, as well as TNF-α or EET-treated groups, were included in the experimental setup. Subsequently, cells were collected for RNA and protein preparation.

### Quantitative real-time reverse transcription PCR (qRT-PCR)

qRT-PCR was performed as previously described [37, 38]. Total RNA was isolated with TRIZOL (Invitrogen, Carlsbad, CA, USA) and used for cDNA synthesis with the Easy Script First-Strand cDNA Synthesis Super Mix kit (TransGen Biotech, Beijing, China) according to the manufacturer's instructions. PCR reactions were set up in triplicate using Power SYBR Green PCR Master Mix (TransGen Biotech). Reactions were performed on the CFX96 system (Bio-Rad, Hercules, CA, USA) according to the manufacturer's instructions. Target gene expression was normalized to the reference gene GAPDH. Primers are listed in Table 1 (annealing temperature = 60°C).

**Table 1:** Sequences of primers used in the real-time PCR

Name		Sequence(5′–3′)
GAPDH (Rat)	Forward	ACTAACCCTGCGCTCCTG
	Reverse	CCCAATACGACCAAATCAGA
Aggrecan (Rat)	Forward	CTTCCCAACTATCCAGCCAT
	Reverse	TCACACCGATAGATCCCAGA
Col1α1 (Rat)	Forward	CGTGGAAACCTGATGTATGC
	Reverse	GGTTGGGACAGTCCAAGTCT
Col2α1 (Rat)	Forward	CGAGGCAGACAGTACCTTG
	Reverse	TGCTCTCGATCTGGTTGTTC
MMP9 (Rat)	Forward	GCTCATCCTACCCATTGCAT
	Reverse	GCTTCCCTGTCATCTTCAGC
MMP3 (Rat)	Forward	TCCTTGCAATGTGGATGTTT
	Reverse	CGTCCTTGAAGAAATGCAGA

GAPDH: Glyceraldehyde 3-phosphate dehydrogenase; Col1α1: Type I collagen (Col I); Col2α1: Type II collagen (Col II); MMP1: Matrix metallopeptidase-1; MMP3: Matrixmetallopeptidase-3; MMP9: Matrix metallopeptidase-9.

### Western blot analysis

Immunoblot analysis was performed in NP cells as described previously [37, 38]. The primary antibodies included mouse anti-GAPDH and mouse anti-β-actin (BOSTER, Wuhan, China). Antibodies specific to phospho-Akt (Ser-473), Akt, phospho-ERK1/2 (Thr202/Tyr204), ERK, phospho-JNK (Thr183/Tyr185), JNK, phospho-p38 (Thr180/Tyr182), and p38 were purchased from Cell Signaling (Boston, MA, USA). Rabbit monoclonal antibodies against Agg, Col I, Col II, MMP3, and MMP9 were purchased from Abcam (Cambridge, MA, USA). Secondary antibodies included goat anti-rabbit IgG-horseradish peroxidase (HRP; sc-2004; Santa Cruz Biotechnology, Santa Cruz, CA, USA) and donkey anti-goat IgG-HRP (sc-2020; Santa Cruz Biotechnology). Immunolabeling was detected using the ECL reagent (BOSTER, Wuhan, China). Protein bands were captured using ChemiDoc™ XRS+ System with Image Lab™ Software (Bio-Rad). Immunoblots were quantified with ImageJ software. Anti-ACTB and anti-GAPDH antibodies were used as loading controls.

### Disc puncture procedure

Rats (*n* = 40) were divided randomly into four groups (*n* = 10 per group): control (non-punctured control), DMSO-treated punctured (vehicle treated), punctured (not treated with either EET or DMSO), and EET-treated punctured groups. Rats were anesthetized using diethyl ether. We used X-rays to identify and mark the target segment before puncture. Each tail was punctured percutaneously at the intervertebral disc between the coccygeal vertebrae with a 21-gauge needle as previously described [39]. After marking the level of the target segments by fluoroscopy, the needle was positioned at the center of the disc parallel to the endplates through the skin and AF into the NP. The tip of the needle was driven to the center of the NP at a depth of 5 mm from the surface of the skin. X-rays were then taken to ensure proper positioning. Subsequently, the needle was turned 360 degrees and left in this position for 30 seconds before removal [40]. In the control group, only the skin was punctured. Following needle puncture, EET (0.5 μg/kg per day) or an equivalent volume of vehicle (low concentrations of DMSO) was slowly injected into the punctured NP. The dose of EET for injection was determined according to the results of previous studies [9, 10, 41]. The treatment was given as 10 doses over a period of 15 days (on postoperative days 0, 1, 2, 3, 4, 6, 8, 10, 12, and 14). To minimize injection injury that might lead to disc degeneration, a micro-syringe attached to a 31-gauge needle was used, and the injection volume was 2 μl [40, 42, 43].

### Radiographic analysis and magnetic resonance imaging

Radiographs and MRI scans of all tails were taken before puncture and 4 weeks afterward. The rats under general anesthesia were settled in a GE X-ray System (GE Mammography DMR Bucky 18 × 24, GE Healthcare, Little Chalfont, UK) in the prone position with their tails straight and secured directly parallel to the axis of the body. Radiographs were taken at a collimator-to-film distance of 66 cm, an exposure of 63 mAs, and a penetration power of 65 kV. Disc heights were measured using the digital radiographs and ImageJ image processing software. The average intervertebral disc height was represented as the DHI and was calculated as described [42, 43].

Magnetic resonance image (MRI) scans were performed using a 7 T MR scanning system (Bruker BioSpin, Rheinstetten, Germany). T2-weighted sagittal sections were rendered using the following settings: fast spin-echo sequence with a time to repetition of 2000 ms and a time to echo of 36 ms; slice thickness = 1 mm; interslice gap = 1 mm; matrix = 256; field of view = 50 mm; and number of averages = 2. A 60-mm volume resonator and a 2-cm diameter surface receive coil were used to maximize image resolution and quality. We analyzed MRI sections of the rat tail spine using a four-grade degeneration grading system adapted from the classification method proposed by Pfirrmann et al. [17].

### Histological analysis

At 4 weeks following surgery, all the rats were euthanized, and the segments were harvested for histological examination. Whole discs with adjacent vertebrae were dissected carefully to avoid injury to the discs, and the spines were fixed in 10% neutral buffered formalin and decalcified using Cal-Ex decalcifying solution HCL (Fisher Scientific, Fairlawn, NJ, USA). Specimens were then rinsed under running tap water and transferred to 75% ethanol. Subsequently, they were embedded in paraffin. Serial mid-sagittal slices from the mid-level of the intervertebral disc were cut at 5 μm thickness and stained with H&E and alcian blue stains [43]. Histological images were analyzed under a microscope. The extent of disc degeneration was graded using a semi-quantitative method, as previously described [39]. Histological sections were graded by blind observers using the system established in previous studies [42].

### Statistical analysis

MRI and histological assessments were conducted by two independent, blind, and experienced observers. Data were presented as the mean of the two evaluations. One observer reassessed images more than 2 weeks after the first evaluation. The Kruskal-Wallis test was used to analyze differences in the T2 signal intensities and in the histological scores of the discs. One-way analysis of variance (ANOVA) was used to analyze differences in disc height. Experiments were performed at least three times. Data were represented as means ± SD. Student's *t*-test was used for comparisons between two groups. ANOVA was used for comparisons involving more than two groups. Statistical significance was set at *p < 0.05. The SPSS version 17.0 software (SPSS, Inc., Chicago, IL, USA) was used for all statistical analyses.
